# Local tree cover predicts mosquito species richness and disease vector presence in a tropical countryside landscape

**DOI:** 10.21203/rs.3.rs-3954302/v1

**Published:** 2024-02-19

**Authors:** Johannah E. Farner, Meghan Howard, Jeffrey R. Smith, Christopher B. Anderson, Erin A. Mordecai

**Affiliations:** Stanford University; Stanford University; Princeton University; Planet Labs; Stanford University

**Keywords:** mosquitoes, Culicidae, community assembly, tree cover, Aedes albopictus, biodiversity

## Abstract

**Context:**

Land use change drives both biodiversity loss and zoonotic disease transmission in tropical countryside landscapes. Developing solutions for protecting countryside biodiversity, public health, and livelihoods requires understanding the scales at which habitat characteristics such as land cover shape biodiversity, especially for arthropods that transmit pathogens. Evidence increasingly shows that species richness for many taxa correlates with local tree cover.

**Objectives:**

We investigated whether mosquito species richness, community composition, and presence of disease vector species responded to land use and tree cover - and if so, whether at spatial scales similar to other taxa.

**Methods:**

We paired a field survey of mosquito communities in agricultural, residential, and forested lands in rural southern Costa Rica with remotely sensed tree cover data. We compared mosquito community responses to tree cover surrounding survey sites measured across scales, and analyzed community responses to land use and environmental gradients.

**Results:**

Tree cover was positively correlated with mosquito species richness, and negatively correlated with the presence of the common invasive dengue vector *Aedes albopictus*, particularly at small spatial scales of 80 – 200m. Land use predicted community composition and *Ae. albopictus* presence. Environmental gradients of tree cover, temperature, and elevation explained 7% of species turnover among survey sites.

**Conclusions:**

The results suggest that preservation and expansion of tree cover at local scales can protect biodiversity for a wide range of taxa, including arthropods, and also confer protection against disease vector occurrence. The identified spatial range of tree cover benefits can inform land management for conservation and public health protection.

## Introduction

Although land use change is a leading cause of loss and endangerment of both biodiversity and ecosystem services (e.g., Sala, 2000; Pereira et al., 2010; Maxwell et al., 2016; Giam, 2017), human-dominated landscapes also have high potential and a pressing need to contribute to conservation (Norris, 2008; Chazdon et al., 2009). Countryside landscapes characterized by natural habitat remnants patchworked with human residential and agricultural infrastructure are globally dominant, and thus represent both an exceptional threat to and exceptional opportunity for conservation (Norris, 2008; Maxwell et al., 2016). A critical requirement for leveraging the conservation potential of countrysides is understanding which landscape features help to retain biodiversity and biodiversity-associated ecosystem services, such as crop pollination and pest control (Frishkoff et al., 2019). Percent tree cover at small spatial scales of <100m has emerged as a reliable predictor of biodiversity for taxa in Latin American tropical countryside landscapes including birds, non-flying mammals, and bats (Mendenhall et al., 2016). These findings suggest that local tree cover management holds promise as a practicable conservation tool, but major knowledge gaps remain surrounding how local tree cover relates to biodiversity and ecosystem services. A key open question relevant to biodiversity conservation is the extent to which local tree cover correlates with species richness across taxa, particularly for invertebrates. Additionally, because tree cover management may influence human – wildlife contact patterns, developing best practices for this conservation tool requires understanding how tree cover in working landscapes relates to vector-borne and zoonotic disease risk.

The spatial scales at which mosquito communities (family Culicidae) respond to tree cover present a knowledge gap relevant to both biodiversity and human health. In addition to playing roles as prey, predators, and detritivores in aquatic and terrestrial food webs (Addicott, 1974; Heard, 1994; Daugherty, Alto and Juliano, 2000; Poulin, Lefebvre and Paz, 2010), several mosquito species are important vectors of wildlife and human diseases including malaria, dengue, chikungunya, Zika, yellow fever, West Nile fever, and arboviral encephalitis (Garmendia, Van Kruiningen and French, 2001; Lemon, 2008; LaPointe, Atkinson and Samuel, 2012; World Health Organization, 2020). Mosquito community composition and abundance are fundamental to when, where, and how widely such diseases are transmitted (reviewed in Franklinos et al., 2019). Landscape context is likely to have multifaceted effects on mosquito community composition through its influence on biotic and abiotic conditions that affect mosquito life cycles. Differences in temperature, habitat structural complexity, and biotic context associated with tree cover are all likely to affect the presence and abundance of mosquito species that vary in their thermal niches, aquatic breeding habitat requirements, and preferred groups of vertebrates for blood meals (Laird, 1988; Gutman et al., 2004; Mordecai et al., 2019; Prevedello et al., 2019). Indeed, previously documented shifts in mosquito communities across habitats show associations between forest conversion and low mosquito biodiversity, and suggest that high rates of land use change and active invasions by major vector species are together reshaping mosquito communities in ways that increase disease risk (Ferraguti et al., 2016; Meyer Steiger, Ritchie and Laurance, 2016; Burkett-Cadena and Vittor, 2018; Chaves et al.,2021). Determining the spatial scales at which tree cover shapes mosquito communities is a critical next step towards understanding how tree cover in countryside landscapes can be managed to balance biodiversity conservation, public health, and economic needs.

The global range expansions of the dengue vectors *Aedes aegypti* and *Aedes albopictus* exemplify ongoing rapid change to mosquito communities that may be linked to concurrent changes in both land cover and disease transmission (Rezza, 2012; Kraemer et al., 2015). In Central America, the first suspected dengue outbreaks occurred in the 1600s; both the disease and the historically common introduced vector *Aedes aegypti* and dengue were eradicated in the 1940s; and both vector and virus were reintroduced in the 1970s (Brathwaite Dick et al., 2012). Dengue in this region has increased dramatically in recent decades: from 378,469 cases reported between 1990 and 2000 to 1,338,330 cases reported between 2007 and 2017 (WHO, 2021). Concurrent with this increase, *Ae. albopictus* was introduced to and spread throughout Central America (Benedict et al., 2007). However, how *Ae. albopictus* colonization relates to dengue in this region is unknown, because its distribution in its new range is poorly documented, and its role in disease transmission must be disentangled from that of *Ae. aegypti*(Rezza, 2012; Kraemer et al., 2015). Globally, *Ae. albopictus* and *Ae. aegypti* are predominantly associated with rural and urban human settlements, respectively, suggesting that *Ae. albopictus* and its responses to tree cover may play particularly important roles in shaping disease risk in countryside landscapes where dengue can be prevalent (Braks et al., 2003; Tsuda et al., 2006).

The countryside of Costa Rica is an ideal system in which to study relationships between land cover, mosquito community characteristics, and disease vector occurrence because this system is the subject of intensive long-term research into links between landscape context, biodiversity, and ecosystem services (Burkett-Cadena and Vittor, 2018). This presents a unique opportunity to improve scientific understanding of mosquito community assembly, and resulting disease risk, in comparison with a rich understanding of other taxonomic diversity and ecosystem services. This is particularly relevant to this region because mosquito-borne diseases, including dengue virus, impose a substantial public health burden. *Aedes aegypti* is considered the primary dengue vector in Costa Rica, but the ongoing, patchily described invasion by the globally important vector species *Aedes albopictusis* potentially reshaping disease risk (Troyo et al., 2006; Calderon-Arguedas *et al.*, 2010; Calderón-Arguedas et al., 2015; Rojas-Araya et al., 2017). Finally, a pioneering national Payment for Ecosystem Services program offers a well-established venue for translating research findings into land management policy, and evidence for additional ecological and human health benefits of local tree cover may help motivate enforcement of an existing law that protects riparian buffer zones up to 50m around rivers, which is expected to increase access to clean water for vulnerable populations, and pertains to a spatial scale at which tree cover has previously been shown to protect biodiversity for other taxa (Sánchez-Azofeifa et al., 2007; Langhans et al., 2022).

Here, we combine field observations of mosquito community composition in forested, agricultural, and residential settings in a rural area of southern Costa Rica with remotely-sensed land cover data in order to ask: (Q1) Does mosquito species richness decline with declining tree cover, consistent with patterns previously observed for a wide range of taxa in a tropical countryside landscape? (Q2) Is the presence of *Aedes albopictus*, a key mosquito vector of human disease, negatively correlated with tree cover? (Q3) How does the spatial scale at which tree cover is observed affect the answers to (Q1) and (Q2)? We additionally ask (Q4) How do mosquito communities respond to environmental gradients versus land use types? Based on evidence that species richness for birds, non-flying mammals, bats, reptiles, and amphibians, and plants increases with tree cover (Mendenhall et al., 2014, 2016), we hypothesize that as local tree cover increases, (H1) mosquito species richness increases, (H2) the presence of human-associated *Aedes albopictus* decreases and (H3) these relationships are strongest at spatial scales of < 100 m. We additionally hypothesize that (H4) shifts in mosquito community composition along tree cover gradients are reflected in distinct forest and residential mosquito species assemblages that are bridged by intermediate agricultural assemblages. By improving scientific understanding of how landscape context influences mosquito community assembly in Costa Rica, the results of this study contribute to a more general understanding of links between land cover, biodiversity, and ecosystem services through the lens of entomological factors that influence disease risk.

## Methods

### Study area

**Figure 1:**
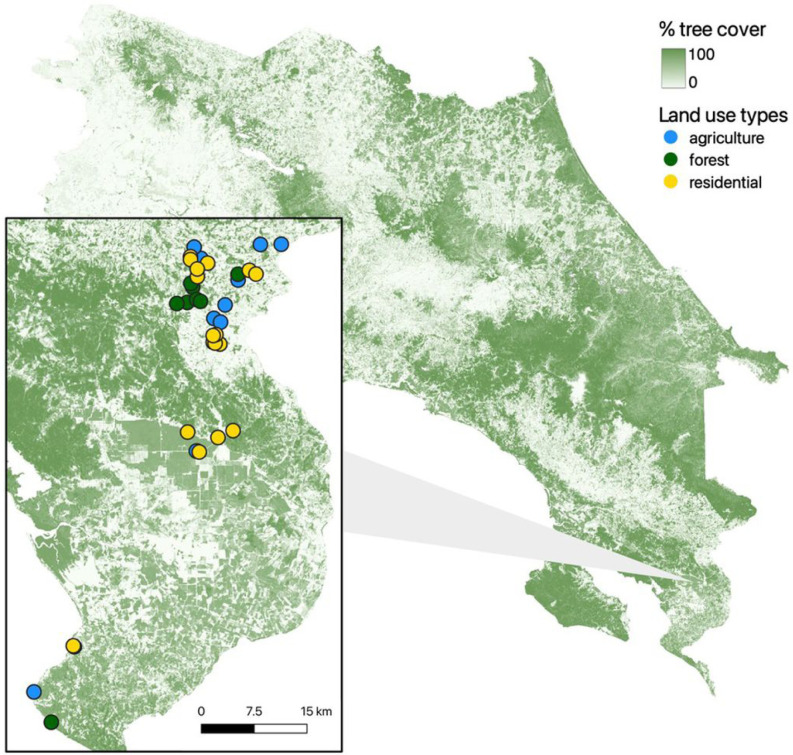
Tree cover and land use varied across study sites in Costa Rica. Darker green indicates higher percent tree cover remotely observed at 30 m resolution. Inset shows study sites, categorized by land use type. Blue, green, and yellow points denote agricultural, forest, and residential land uses, respectively.

This study was conducted in the cantons of Coto Brus, Corredores, and Golfito (8°43’14”N, 82°57’20”W)_J_ located in the southern Puntarenas region of Costa Rica along the border with Panama (Fig. 1). The region ranges from coastal lowland tropical rainforest (0 m above sea level) to high elevation cloud forest (1500 m above sea level) and has distinct wet and dry seasons. The study area is predominately composed of rural communities surrounded by agriculture interspersed with forest patches, and also includes the protected Las Cruces forest reserve. Dengue reintroduction in Costa Rica was first reported from the Puntarenas region in 1993, where the disease has since been endemic (World Health Organization, 1994; Troyo et al., 2009). *Ae. aegypti* is common and *Ae. albopictus* has a growing presence in this area (Troyo et al., 2008; Rojas-Araya et al., 2017).

### Study sites

With landowner permission, we accessed 37 sites located in San Vito, Sabalito, Copa Buena, Ciudad Neilly, and Pavones (Fig. 1). The sites represented three broad land use classes that were determined onsite by the survey team: residential (N = 17), agricultural (N = 12), and forest (N = 8) (Fig. 1). Residential sites included urban and peri-urban areas; agricultural sites included coffee plantations, an oil palm plantation, a pine plantation, one mixed agricultural field, and one pasture; forested sites included primary and secondary forest edges, interiors, and fragments.

### Environmental variables

To quantify percent tree cover, we used a 30 m resolution map of tree cover in Costa Rica created by Echeverri et. al (2022) from multi-sensor satellite observations and fine-scale tree cover maps (Fig. 1). From this map, we calculated percent tree cover at different spatial scales surrounding each site using the R package “raster” (Hijmans et al., 2015). Specifically, we began by calculating percent tree cover within a radius of 30 m; we then increased this radius by increments of 10 m up to 200 m, and by increments of 50 m for radii between 200 m and 1000 m (following Mendenhall et. al, 2014). To account for mosquito interspecific variation in sensitivity to thermal conditions (Mordecai et al., 2019), which could affect observed relationships between land cover and mosquito community characteristics along the 1500 m elevational gradient surveyed, we additionally extracted mean annual temperature data from 1970–2000 for each study site from the WorldClim 1 km^2^ resolution mean annual temperature dataset (Fick and Hijmans, 2017).

### Sample collection

We trapped mosquitoes at all sites twice between June 19 and August 9, 2017, excepting the sites in Pavones, which were trapped once. At each site, we placed a total of four traps overnight for a 12–16 hour period within an area of 30 m in radius: one unlighted CDC trap baited with carbon dioxide produced by a mixture of Fleischmann’s Dry Active Yeast, household refined sugar, and water; one BG Sentinel baited with a BG-Lure and octanol; and two BG-GAT traps furnished with yellow sticky cards, corn oil, and a mixture of water and local leaf litter. Trap locations within sites were chosen per BioGents recommendations, and square metal frames covered in large black plastic bags were placed over BG Sentinel and BG-GAT traps for protection from rainfall. To supplement the overnight trapping, consistent members of the field team carried out 20 minutes of direct aspiration at each site during each trapping session.

### Mosquito identification

Trained personnel identified, sexed, and counted any *Ae. albopictus* and *Ae. aegypti* mosquitoes collected at each site. All other mosquitoes were counted, stored, and transported to Stanford University for molecular identification. We extracted and amplified DNA from the mitochondrial CO1 gene from pooled samples of mosquitoes from each site using MyTaq RedMix (Meridian Bioscience, Cincinnati, OH), following the protocol provided by the manufacturer. Amplified DNA was sequenced via Illumina next-generation sequencing, with samples containing *Aedes albopictus* and *Culex tarsalis* DNA as positive controls. We used the R package “dada2” to filter and trim the DNA sequences to 473 bp, with a minimum overlap of 20 bases and a maximum of 5 expected errors (Callahan et al., 2016). We estimated taxonomic placement for the sequenced mosquitoes by using the R packages “Biostrings” and “DECIPHER” to group DNA sequences into operational taxonomic units (OTUs, henceforth referred to as species) of 97% sequence similarity, and comparing representative sequences for each species to the BOLD and GenBank database records (Altschul et al., 1990; Ratnasingham and Hebert, 2007; Wright, 2016; Pagès et al., 2022). Species were identified based on top matches with sequence similarity > 97% (Hardulak et al., 2020). When sequence similarity to the top match was < 97%, a higher level of taxonomic identification (e.g., genus) was assigned based on placement within a phylogenetic tree of the BOLD database sequences.

### Statistical analyses

We described mosquito communities in terms of species richness and species composition by combining presence data from the morphologically identified *Aedes* data and the sequencing data. To quantify relationships between species richness and percent tree cover, we used generalized linear models (GLMs) with negative binomial error corrections for overdispersion and mean-centered independent variables. To assess the spatial scales at which tree cover best predicted species richness, we compared AIC values for GLMs that included percent tree cover surrounding each site calculated at radii ranging from 30 m to 1000 m. We used binomial logistic regression to analyze relationships between *Ae. albopictus* disease vector presence/absence and percent tree cover across spatial scales. For the 1000 m spatial scale where climate data were available, we additionally assessed the relative influence of mean annual temperature on species richness and *Aedes* vector presence with GLMs including mean annual temperature and its interaction with tree cover.

To compare species richness and *Aedes* vector presence between forest, agricultural, and residential land uses, we used Kruskal-Wallis tests with Bonferroni p-value adjustments to account for multiple comparisons.

To compare species composition among land use types and along environmental gradients, we first calculated the Jaccard coefficient of community similarity for each pair of sites for use in statistical tests and ordination. We then tested for differences in community similarity among land uses with permutational analysis of variation (PERMANOVA), first for all land use types, and then with pairwise adonis functions. Because PERMANOVA is sensitive to heterogeneity in dispersion among groups (Anderson and Walsh, 2013), we additionally tested whether dispersion differed among land use types using Tukey’s Honest Significant Differences method with betadisper() calculations of group average distances to the median. To visualize community similarity across land uses, we used non-metric multidimensional scaling (NMDS). All the above analyses of compositional similarity among land uses were run using the R package “vegan” (Oksanen et al., 2013); for the pairwise PERMANOVA analyses, we used the R package “ecole” which provides wrapper functions for “vegan” (Smith, 2022). Finally, to quantify compositional turnover along environmental gradients of tree cover, mean temperature, elevation, and geographic distance, we used the R package “gdm” for generalized dissimilarity modeling (GDM), a form of nonlinear matrix regression that is robust to collinearity (Fitzpatrick et al., 2022). As above, we compared GDM models that incorporated tree cover at radii ranging from 30–1000 m to identify the spatial scale at which tree cover best explained compositional turnover.

All analyses were performed in R version 4.2.1. In addition to the R packages cited above, we used the packages “tidyverse” (Wickham et al., 2019), “cowplot” (Wilke et al., 2019), “MASS” (Ripley et al., 2013), “interactions” (Long, 2019), “gridExtra” (Auguie et al., 2017), and “reshape2” (Wickham, 2007) for data analysis and figure generation.

## Results

Across sites, tree cover ranged from 0–100% (mean = 27.9%, SD = 34.8%) at the smallest spatial scale we considered, a 30 m radius. Surrounding tree cover within a 1000 m radius, the largest spatial scale considered, ranged from 8.2–74.8% (mean = 33.4%, SD = 20.3%). The lowest and highest site elevations were 12 m and 1451 m above sea level, respectively (mean = 776 m, SD = 473.2 m). Mean annual temperature ranged from 18.8 to 26.4 °C (mean = 22.6 °C, SD = 2.3 °C).

**Figure 2:**
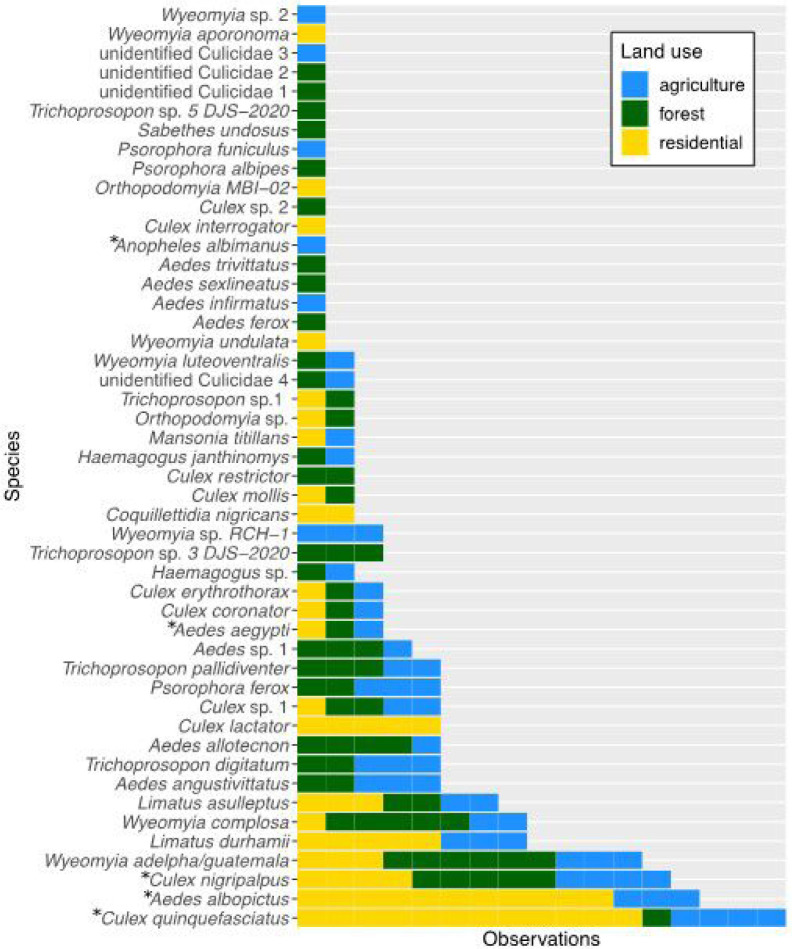
The commonness of 48 observed mosquito species varied both overall and among land use types. Blue bars show the number of presences observed for each species in agricultural sites, green bars show forest sites, and yellow bars show residential sites. Asterisks indicate species known to vector human diseases.

A total of 1,283 mosquitoes representing 48 species in 13 genera were collected from 34 sites (Fig. 2). The number of mosquitoes collected at a site ranged from 0 to 244 (mean = 35, SD = 64). Of these, 108 individuals from 12 residential and four agricultural sites were morphologically identified as *Ae. albopictus*, and 5 individuals from single sites within each land use category were identified as *Ae. aegypti. Ae. albopictus* DNA was detected in the pooled samples of molecularly identified mosquitoes from 7 sites where *Ae. albopictus* individuals were also morphologically identified. The five most common species were *Culex quinquefasciatus, Ae. albopictus, Cx. nigripalpus, Wyeomyia adelpha/Guatemala*, and *Limatus durhamii* (Fig. 2, Table S1).

Site-level species richness ranged from one to 19 (mean = 5.2, SD = 4.1). Overall species counts for forest, agricultural, and residential land uses were 33, 29, and 21, respectively. Ten species (21 %) were observed in all three land uses. Nineteen species (40%) were shared among forest and agricultural land uses, 13 species (27%) were shared among agricultural and residential land uses, and twelve species (25%) were shared among forest and residential land uses (Fig. 2, Table S1). Eleven species (23%) were found only in forested settings, six species (13%) were found only in agricultural settings, and six species (13%) were found only in residential settings (Fig. 2, Table S1). Two species, *Ae. albopictus* and *Culex quinquefasciatus*, were common (observed at > 50% of sites) in residential settings, no species were common in agricultural settings, and three species-*Culex nigripalpus, Wyeomyia complosa*, and *Wyeomyia adelpha/guatemala—were* common in forested settings (Table S1).

At least five of the mosquito species observed are known vectors of human diseases. Three of these— the dengue and chikungunya virus vector *Ae. albopictus* (present at 15 sites) and the St. Louis Encephalitis virus vectors *Cx. quinquefasciatus* (present at 17 sites) and *Cx. nigripalpus* (present at 13 sites)—were the three most frequently observed species (Fig. 2, Table S1) (Reisen, 2003; Simmons et al., 2012). Rarely observed vector species included the dengue, chikungunya, yellow fever, and Zika virus vector *Ae. aegypti* (present at three sites spanning all three land use types) and the malaria vector *Anopheles albimanus* (present at one agricultural site) (Fig. 2, Table S1) (Zimmerman, 1992; Simmons et al., 2012). In contrast to *Ae. aegypti, Cx. nigripalpus*, and *Cx. quinquefasciatus*, which were observed in all land use types, *Ae. albopictus* was observed only in residential and agricultural settings associated with intensive human modification (Fig. 2, Table S1).

**Figure 3:**
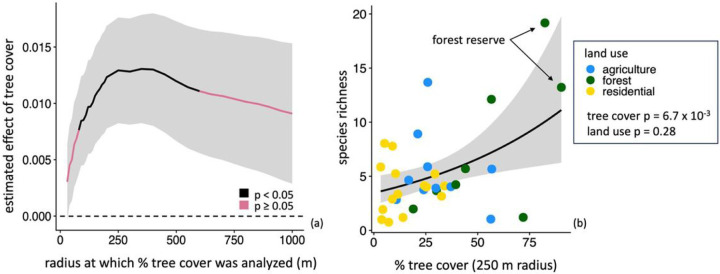
Species richness is correlated with tree cover surrounding survey sites for radii between 80 m and 600 m. (a) The estimated effect of surrounding tree cover calculated across spatial scales on species richness. Radii where the relationship between tree cover and species richness is significant (p < 0.05) are shown in black. Values above and below the dashed line are positive and negative, respectively, (b) Species richness increases with percent tree cover at the 250 m radius: the scale identified as having the strongest effect. Land use (colored points) and species richness are not significantly correlated. The site with the highest species richness and the site with the highest surrounding tree cover were both located in the Las Cruces forest reserve (arrows). In both panels, gray shading shows +/−1 SE.

Mosquito species richness was explained by tree cover, but not by land use type. Comparisons of GLMs using tree cover calculated for radii ranging between 30 m and 1000 m surrounding each site indicated that species richness was positively correlated with tree cover at radii between 80 m and 600 m, and tree cover at a 250 m radius had the largest effect size (estimated effect = 1.29 × 10^−2^, SE = 4.75 × 10^−3^, z-value = 2.71, p-value = 6.65 × 10^−3^) (Fig. 3a, Table S2). At the 1000 m spatial scale where both tree cover and climate data were available, the interaction between tree cover and mean annual temperature had a significant effect on species richness (estimated effect = 7.87 × 10^−3^, SE = 3.65 × 10^−3^, z-value = −2.16, p-value = 3.11 ×^10−^2 (Table S3). Specifically, at high temperatures, species richness was low even when tree cover was high (Figure S1). By contrast, Kruskal-Wallis test results indicated that species richness did not differ significantly among forested, agricultural, and residential sites (chi-squared = 2.60, df = 2, p-value = 28) (Fig. 3b). Notably, the highest species richness was observed at a site in the Coto Brus forest reserve (Fig. 3b).

**Figure 4:**
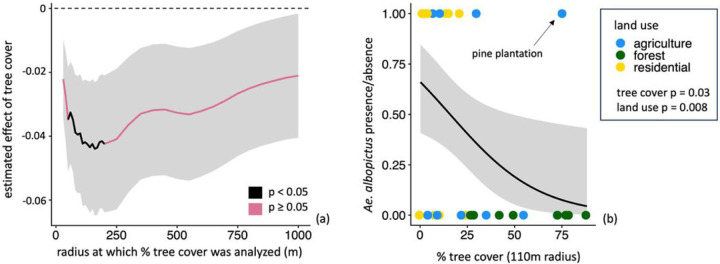
Aedes albopictus is most likely to be found at low tree cover levels and in residential settings. (a) The estimated effects of tree cover across spatial scales on **Ae. albopictus** presence, where black lines indicate statistically significant (p < 0.05) effects and pink lines indicate nonsignificant (p > 0.05) relationships. Values above and below the dashed line are positive and negative, respectively, (b) Land use type (colored points), percent tree cover and Ae. albopictus presence/absence at the highest-significance 110 m radius. In both panels, gray shading shows +/−1 SE.

From the *Aedes* disease vector survey, we present results only for *Ae. albopictus* because observations of *Ae. aegypti* were insufficient for statistical analysis. Both tree cover and land use type predicted *Ae. albopictus* presence. Comparisons of GLMs using tree cover calculated for radii ranging between 30 m and 1000 m surrounding each site indicated that *Ae. albopictus* presence was negatively correlated with tree cover at radii between 50 m and 200 m, and was best explained by tree cover at a 110 m radius (estimated effect = −4.23 × 10^−2^, SE = 1.96 × 10^−2^, z-value = −2.16, p-value = 3.08 × 10^−2^) (Fig. 4a, Table S3). At the 1000 m spatial scale where we additionally assessed the influence of climate, *Ae. albopictus* presence was negatively correlated with tree cover and positively correlated with temperature (tree cover estimated effect = −7.76 × 10^−2^, SE = 3.74 × 10^−2^, z-value = −2.07, p-value = 3.81 × 10^−2^; mean annual temperature estimated effect = 6.62 × 10^−1^, SE = 3.35 × 10^−1^, z-value = 1.97, p-value = 4.814 × 10^−2^) (Table S5). Land use type also predicted *Ae. albopictus* presence (Kruskal-Wallis chi-squared = 9.58, p-value = 8.311 × 10^−3^). Specifically, *Ae. albopictus* was significantly more likely to be observed in residential settings (present at 13/17 sites) than in forested settings (present at 0/8 sites) (Kruskal-Wallace chi-squared = 9.03, Bonferroni-adjusted p-value = 7.98 × 10^−3^), and its presence in agricultural settings (present at 4/12 sites) did not differ significantly compared to either residential (Kruskal-Wallace chi-squared = 2.41, adjusted p-value = 3.6 × 10^−1^) or forested (Kruskal-Wallace chi-squared = 3.04, adjusted p-value = 2.43 × 10^−1^) settings. Fourteen of the 15 sites where *Ae. albopictuswas* present were surrounded by less than 25% tree cover within a 50 m radius. The only site within a pine plantation was a clear outlier, where *Ae. albopictuswas* present under 85% tree cover (Fig. 4B).

**Figure 5:**
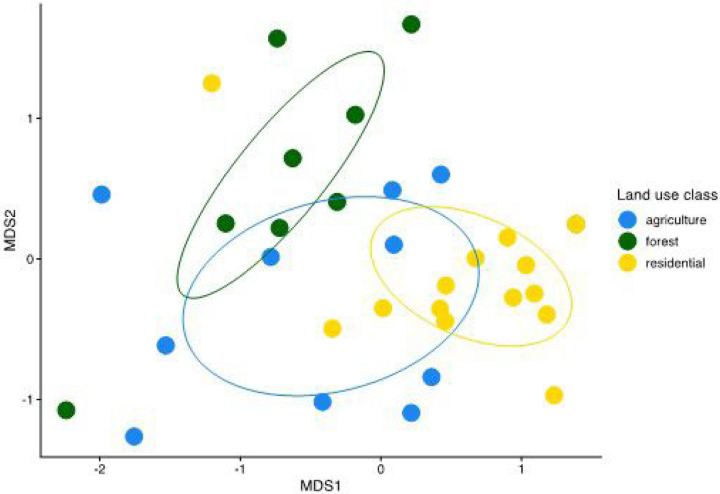
Distinct mosquito communities were observed in forest and residential land uses, while communities in agricultural settings were more variable. NMDS ordination visualization groups sites by community similarity, with colors indicating land use types. Each point represents the community at one study site, and the distance between points is smaller for more similar communities. Ellipses show 95% confidence intervals for ordination of agriculture, forest, and residential mosquito communities.

In contrast to species richness, community composition and dispersion were predicted by land use type. PERMANOVA results comparing all three land uses showed that land use significantly affected community composition (Sum of squares = 2.113, R^2^ = 0.16, F-value = 3.03, p-value = 0.001), and pairwise PERMANOVAs showed that agricultural and forest mosquito communities differed significantly from residential communities (Table 1). Wider dispersion among agricultural compared to residential mosquito communities (average distance to the median: agriculture = 0.612, forest = 0.572, residential = 0.497; Tukey test adjusted p-values: residential – agricultural = 0.0422, residential - forested = 0.303, forested – agricultural = 0.736) likely contributed to the community dissimilarity detected between these land uses (Anderson and Walsh, 2013) (Fig. 5). NMDS visualization of communities grouped by land use type suggests that more variable agricultural communities bridge relatively distinct forest and residential communities (NMDS stress = 0.12), in agreement with the statistical test results (Fig. 5, Table 1). The wider variation among agricultural sites is also evident from the species observation table: no single species was observed at more than 1/3 of all agricultural sites, whereas *Ae. albopictus* and Unidentified Culicidae 1 were both observed at > 70% of residential sites, and *Culex nigripalpus, Wyeomyia adelpha/guatemala*, and *Wyeomyia complosa* were each observed at > 60% of forested sites (Table S1).

**Figure 6:**
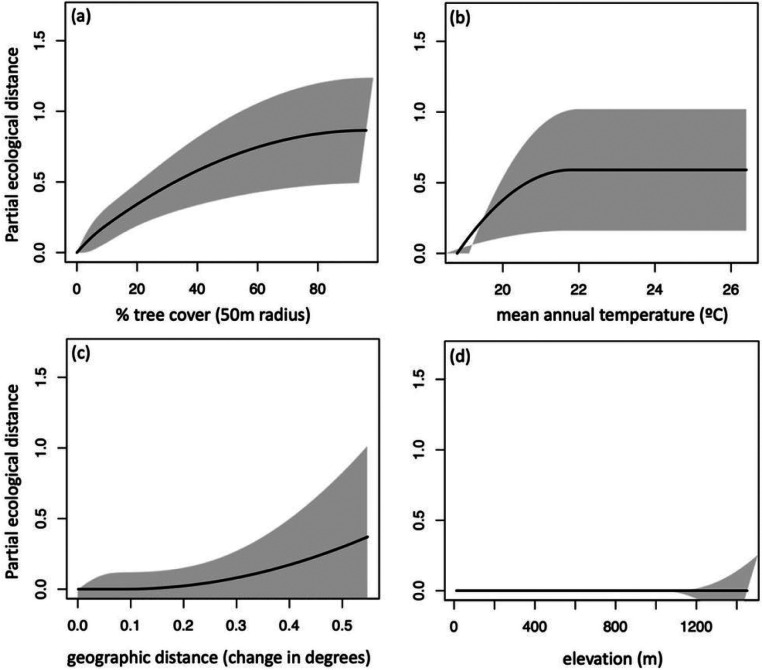
Generalized dissimilarity modelling (GDM) of community dissimilarity indicated that (a) percent tree cover and (b) mean annual temperature explained 7% of deviation from the null. (c) geographic distance and (d) elevation did not significantly contribute to community turnover. In each panel, the x-axis shows the environmental gradient and the y-axis shows the amount of compositional turnover, measured as partial ecological distance. The maximum height the spline reaches on the y-axis indicates the total amount of compositional turnover the gradient is associated with, and the slope shows how the rate of compositional turnover varies along the environmental gradient. The difference in height between any two points along the I-spline corresponds to the modeled contribution of that predictor variable to the difference between those points. Grey shading shows +/−1 standard deviation when 70% of sites are sampled 10 times.

Finally, generalized dissimilarity modeling (GDM) indicated that environmental gradients explained little of the species turnover among sites. Among the spatial scales for which tree cover was calculated, the model using the 50m radius explained the highest amount of species turnover among sites (Table S6). The model that included mean annual temperature, geographic distance, and tree cover at the 50m radius explained 7% of species turnover among sites. Elevation showed no relationship with species turnover. Whereas increasing tree cover was associated with a consistent increase in community turnover, increasing temperature was associated with a steep increase in community turnover up to a plateau around 22 °C (Fig. 6).

## Discussion

We found that local tree cover, but not land use (residential, agricultural, or forest), predicted mosquito species richness: more diverse communities occurred at higher tree cover (supporting H1) (Fig. 3, Table S2). By contrast, community composition was more predictable for forested and residential land uses, and more variable among agricultural sites (supporting H4) (Fig. 5, Table 1). Environmental gradients of tree cover, climate, and geographic distance explained 7.2% of species turnover among sites (Fig. 6, Table S6). *Ae. albopictus* presence varied significantly with both tree cover and land use, but in the opposite direction from mosquito diversity: *Ae. albopictus* occurrence probability increased with lower tree cover and in residential compared to forested sites (supporting H2) (with intermediate probability in agricultural sites; Fig. 4, Table S4). Overall, our results add to support from other taxa for the value of both natural and semi-natural habitat in sustaining biodiversity and ecosystem services—here, in the form of protection against an invasive mosquito that is a major vector of human disease.

### Mosquito diversity

The spatial scales at which tree cover predicted mosquito species richness were small, and comparable to previous findings for other taxa in the same study area, highlighting the disproportionately positive impact of small patches of trees on biodiversity. The radius at which tree cover best predicted species richness was 250 m; by comparison, biodiversity was correlated with tree cover at small spatial scales for non-flying mammals (70 m), bats (50–60 m), birds (30 m), reptiles (50 m), and amphibians (80 m) in the same region of Costa Rica (Mendenhall et al., 2014, 2016; Frank et al., 2017). Although the radius at which mosquito species richness responded most strongly to tree cover was larger compared to previously studied taxa, the spatial scale remained local, and significant effects of tree cover were found at radii as small as 80 m (Fig. 3). Our observation of a positive relationship between species richness and tree cover aligns with those of many other studies of mosquito diversity along land cover gradients in locations including Latin America, Asia, and Europe (e.g., Johnson, Gomez and Pinedo-Vasquez, 2008; Thongsripong et al., 2013; Ferraguti et al., 2016; Chaverri et al., 2018), and clarifies the spatial scales at which tree cover shapes mosquito communities.

Our observation that mosquito community composition was distinct among different land uses is consistent with patterns observed both for other taxa in this system, and for mosquitoes in other regions (Mendenhall et al., 2014, 2016; Meyer Steiger, Ritchie and Laurance, 2016; da Silva Pessoa Vieira et al.,2022). In agricultural settings, relatively high species richness and community similarity with forested settings support the argument that farmlands can contribute substantially to biodiversity conservation (Fig. 5, Table 1) (e.g., Norris, 2008). However, the high proportion of species unique to forest habitats and the high species richness observed inside the large Las Cruces forest reserve also reaffirm the singular importance of forests and protected areas as refugia for biodiversity (Coetzee, Gaston and Chown, 2014; Mendenhall et al., 2016) (Fig. 3, Table S1). Additionally, the compositional variability among agricultural settings and the close community resemblance between some agricultural and residential sites indicate a need for additional research on how mosquito communities respond to specific land uses, crop assemblages, or management practices that can result in similar levels of tree cover (Fig. 5, Table 1). For example, organic farming methods are associated with higher arthropod diversity globally compared to conventional methods, and Kenyan ricelands that rely on natural rather than artificial irrigation have higher mosquito species richness Kenya (Muturi et al., 2006; Lichtenberg et al., 2017). Costa Rican croplands that are less intensively farmed may similarly support greater mosquito biodiversity, a pattern already observed for bird species richness (Hendershot et al., 2020).

Environmental gradients of tree cover and temperature shaped species turnover, but explained only 7% of variance in community composition, suggesting that additional habitat characteristics may play important roles in determining species composition. Such factors might include local microclimates, differences among types of tree cover (e.g., agricultural types, primary versus secondary forest), and/or the presence of vertebrate hosts preferred by different mosquito species. Differences in species abundances and community evenness, which were not quantified here, might also respond more strongly to gradual environmental change than the identities of the species present. However, our result that land use predicts species composition, while land cover predicts diversity, aligns with patterns of abundance-based Dipteran and Culicid diversity observed in the tropical Australian countryside (Smith and Mayfield, 2015; Meyer Steiger, Ritchie and Laurance, 2016).

### Disease vectors

The most frequently observed disease vector, *Ae. albopictus*, was more likely to be observed in sites with lower surrounding tree cover and agricultural or residential land uses, suggesting that rural landscapes with more forest and tree cover may be more resistant to invasion by this species. These observations align with this species’ well-established preferences for taking blood meals from humans and livestock (Niebylski et al., 1994; Richards et al., 2006), and its association with rural, agricultural, suburban, and/or deforested settings in the Americas, Asia, and Africa (Gilotra, Rozeboom and Bhattacharya, 1967; Braks et al., 2003; Young et al., 2017; Camara et al., 2020; Canelas et al., 2023). The 40–200 m distances where tree cover negatively affected *Ae. albopictus* presence fell within the 80–600 m range where tree cover positively affected species richness, suggesting promise for local tree cover management as a means of supporting both public health and biodiversity conservation. Protection against mosquito disease vectors conferred by tree cover may extend beyond *Ae. albopictusto* include at least 16 other significant vectors of human diseases that are favored by deforestation, including *Ae. aegypti*, multiple Anopheline malaria vectors, and the Amazonian malaria vector *Nyssorhynchus darlingi*(Burkett-Cadena and Vittor, 2018; Chaves et al., 2021).

Our finding that *Ae. albopictus* was associated with, but inconsistently observed in, agricultural settings (present in 33% of agriculture sites) (Fig. 4, Table S1) reinforces that agricultural lands have the potential to either harbor or resist invasive species, and suggests vector associations with agricultural subtypes as a key future research direction. In our survey, factors that differentiated the high tree-cover pine plantation and two of the six surveyed coffee plantations as suitable habitat for *Ae. albopictus* are of particular interest (Fig. 4). Understanding vector responses to agriculture is particularly important because this land use is the most likely candidate for local tree cover management in Costa Rica due to its spatial extent, the established Payment for Ecosystem Services program infrastructure for incentivizing landowner forest retention and tree planting, and a previous finding that urban tree cover is correlated with dengue incidence in this region (Sanchez-Azofeifa et al., 2007; Troyo et al., 2009).

However, further research is needed to understand how tree cover management could be applied for such public health protections. For example, future studies should aim to capture the seasonality of vector presence and abundance along tree cover gradients (Calderón-Arguedas et al., 2008; Troyo et al., 2008; Romero-Vega et al., 2023); assess relationships between vector presence and other factors in habitats that have similar tree cover but differ in aspects such as crop type or tree cover geometry; and use reforestation efforts or experimental tree cover additions at relevant spatial scales to test mosquito community responses. In the study region, tree cover responses of *Ae. aegypti* and *An. albimanus* require clarification, as we observed these regionally important vectors too rarely for statistical analysis, and saw unexpected rarity and apparent habitat generalism in the canonically widespread and urban vector *Ae. aegypti* (Fig. 2, Table S1) (Troyo et al., 2006; Caceres et al., 2012). Additionally, the potential disease control benefits of local tree cover should be tested with human pathogen surveillance in field-captured mosquitoes from different environments, and by comparison of disease case data to mosquito community data. In addition to dengue, Zika, and malaria, St. Louis Encephalitis Virus should be considered for surveillance in rural areas where humans and animals live in close proximity, because two potential vectors—*Cx*. *quinquefasciatus* and *Cx. nigripalpus*—were common, and this unmonitored disease is already widespread among both domesticated and wild animal hosts in Costa Rica (Medlin et al., 2016; A. Chaves et al., 2021; Piche-Ovares et al., 2023).

## Conclusions

Overall, our results join a large body of evidence for the importance of local tree cover to maintaining both biodiversity and ecosystem services, adding prevention of disease vector proliferation to a list of benefits including water quality, crop pollination, carbon sequestration, and soil health, among others (Millennium Ecosystem Assessment, 2005; Jose, 2009; Barrios et al., 2018; Frishkoff et al., 2019). Our findings follow patterns observed repeatedly across the globe associating tree cover with higher mosquito species richness and lower disease vector presence, suggesting broad applicability of local tree cover management as a combined vector control and biodiversity conservation method. Further, we shed light on the contexts in which tree cover both increases mosquito species richness and decreases *Ae. albopictus* presence, namely at spatial scales of 80–200m (Figs. 2,3; Tables S2, S4). We also find that at larger spatial scales of 1000m, warm mean annual temperatures increase habitat suitability for *Ae. albopictus* and limit tree cover contributions to species richness, but note that other factors that covary with climate across the study region may contribute to this result (Figure S1, Tables S3, S5). Although the specific mechanisms and characteristics by which tree cover inhibits disease vectors remain unclear, the regularity with which simple measures of local tree cover correlate with both biodiversity and ecosystem services represents a practicable solution for fostering both human and ecosystem health in countryside landscapes.

## Figures and Tables

**Table 1:** PERMANOVA results for community composition compared among land use types. Asterisks indicate p < 0.05.

Land use pair	Sum of squares	F-Value	R^2^	Bonferroni-adjusted p-value
agriculture vs. forest	0.591	1.45	0.0785	0.291 *
agriculture vs. residential	0.788	2.22	0.0846	0.021 *
forest vs. residential	1.56	4.63	0.181	0.003 *

## Data Availability

The data presented in this manuscript are available in the DRYAD repository at https://doi.org/10.5061/dryad.p8cz8w9xg.
